# Alvaxanthone, a Thymidylate Synthase Inhibitor with Nematocidal and Tumoricidal Activities

**DOI:** 10.3390/molecules25122894

**Published:** 2020-06-23

**Authors:** Piotr Maj, Mattia Mori, Justyna Sobich, Joanna Markowicz, Łukasz Uram, Zbigniew Zieliński, Deborah Quaglio, Andrea Calcaterra, Ylenia Cau, Bruno Botta, Wojciech Rode

**Affiliations:** 1 Nencki Institute of Experimental Biology, 3 Pasteur Street, 02-093 Warsaw, Poland; p.maj@nencki.edu.pl (P.M.); j.sobich@nencki.edu.pl (J.S.); z.zielinski@nencki.edu.pl (Z.Z.); 2 Department of Pharmacology, University of Oxford, Mansfield Road, Oxford OX1 3QT, UK; 3 Department of Biotechnology, Chemistry and Pharmacy, Department of Excellence 2018-2022, via Aldo Moro 2, 53100 Siena, Italy; m.mattia79@gmail.com (M.M.); cau.ylenia@gmail.com (Y.C.); 4 Faculty of Chemistry, Rzeszów University of Technology, 6 Powstańców Warszawy Ave, 35-959 Rzeszów, Poland; jmarkowicz@stud.prz.edu.pl (J.M.); luram@prz.edu.pl (Ł.U.); 5 Department of Chemistry and Technology of Drugs, Department of Excellence 2018–2022, Sapienza University of Rome, Piazzale Aldo Moro 5, 00185 Rome, Italy; deborah.quaglio@uniroma1.it (D.Q.); andrea.calcaterra@uniroma1.it (A.C.); bruno.botta@uniroma1.it (B.B.)

**Keywords:** thymidylate synthase, inhibitor, nematocidal activity, cytotoxicity, xanthones

## Abstract

With the aim to identify novel inhibitors of parasitic nematode thymidylate synthase (TS), we screened in silico an in-house library of natural compounds, taking advantage of a model of nematode TS three-dimensional (3D) structure and choosing candidate compounds potentially capable of enzyme binding/inhibition. Selected compounds were tested as (i) inhibitors of the reaction catalyzed by TSs of different species, (ii) agents toxic to a nematode parasite model (*C. elegans* grown in vitro), (iii) inhibitors of normal human cell growth, and (iv) antitumor agents affecting human tumor cells grown in vitro. The results pointed to alvaxanthone as a relatively strong TS inhibitor that causes *C. elegans* population growth reduction with nematocidal potency similar to the anthelmintic drug mebendazole. Alvaxanthone also demonstrated an antiproliferative effect in tumor cells, associated with a selective toxicity against mitochondria observed in cancer cells compared to normal cells.

## 1. Introduction

Thymidylate synthase (TS; EC 2.1.1.45) catalyzes the reductive methylation of deoxyuridine monophosphate (dUMP) by N^5,10^-methylenetetrahydrofolate (meTHF) to generate thymidylate (dTMP) and dihydrofolate. Being the sole de novo source of dTMP in animal cells [1], the enzyme is consequently a target of antitumor, antiviral, antifungal, and antiprotozoan chemotherapy [2,3,4,5,6,7,8,9].

Parasitic nematodes belong to the most common infectious agents, and the majority of infections affect developing countries [10,11,12]. In our previous studies, we were interested in *Trichinella spiralis*, which is responsible in both developing and developed countries for a serious disease, i.e., trichinellosis [13], and a free-living nematode *Caenorhabditis elegans*, which is often used as a model organism in parasitological studies [14,15,16]. Of particular interest was a high TS-specific activity present throughout the developmental cycles of the two nematode species, including their developmentally arrested forms (lacking cell proliferation and thus expected to show TS activity either low or none at all), including *T. spiralis* infective muscle larvae [16,17,18,19] and *C. elegans* dauer larvae [17], the latter corresponding to developmentally arrested infective larvae of parasitic nematodes [14]. It pointed to the high TS level as a result of an unusual cell cycle regulation, leading to a long-term cell cycle arrest, in the developmentally arrested larvae (discussed in Reference [17,18]). Although TS protein in those larvae is probably catalytically irrelevant (no DNA synthesis), it may play a regulatory role in view of the enzyme’s certain non-catalytic activities, including capacity to bind mRNA (its own and some others) and inhibit translation, with potential regulation of several cellular genes [19,20], as well as an oncogene-like activity [21]. Thus, in view of the latter, a possibility to selectively interfere with nematode TS catalytic/non-catalytic activities could be applied not only in an attempt to kill a parasite but also to study the physiological significance of the high expression of TS in nematodes’ cells, particularly in their developmentally arrested larvae.

The present study was aimed at seeking new TS inhibitors within the in-house library of natural compounds and their derivatives (around 1000 compounds) organized and maintained by the group of Professor Bruno Botta of Sapienza University of Rome. Of particular interest was a possibility of inhibition of parasitic nematode TS. Thanks to the availability of a model nematode TS X-ray crystallographic structure, candidate compounds potentially capable of enzyme binding/inhibition were identified by means of a structure-based virtual screening of the above library. In an attempt to make the best use of the results of the screen and considering a strong conservation of the enzyme protein [5], the selected compounds, confirmed to be TS inhibitors, were tested not only as antinematode but also as antitumor agents. Therefore, the tested properties included (i) potential to inhibit the reaction catalyzed by TSs of different specific origin, (ii) toxicity to a nematode parasite model (*C. elegans* grown in vitro), (iii) potential to inhibit normal human cell growth, and (iv) antitumor activity affecting human tumor cells grown in vitro.

## 2. Results

### 2.1. Chemical Library and Virtual Screening

The in-house library of natural product contains around 1000 small molecules isolated, purified, and characterized mostly from plants used in the traditional medicine of South America countries, as well as a number of chemical derivatives. The library owns a significant chemical diversity and was already used as source of hit and lead compounds in previous drug discovery projects [22,23,24,25,26,27]. To pursue the aim of identifying potential TS inhibitors, here, 865 compounds from the library were screened against the crystallographic structure of *C. elegans* TS in complex with 2′-deoxyuridine-5′-monophosphate (dUMP) and the small molecule inhibitor Tomudex (PDB ID: 4IQQ). Docking simulations were carried out with FRED (OpenEye scientific software) on the Tomudex binding site, after removing the coordinates of Tomudex and co-crystallized water molecules from the receptor structure. Docking results were then sorted according to the FRED score, which is calculated by the Chemgauss4 function, while the shortlist of compounds to select for biological studies was finalized by a combination of score, visual inspection, and chemical diversity. This operation led to the selection of 20 natural compounds as putative TS inhibitors (Table 1).

### 2.2. Predicted Properties of Compounds From the In-House Library

The majority of compounds are likely to exhibit favorable ADME/tox properties. Apart from agnuside and leucospectoside A, all remaining compounds follow the Lipinski Rule of 5 [28] with either one or no violations. Similarly, agnuside and leucospectoside A are consistently among compounds with the most violations of other sets of medchem rules, e.g., Veber rules [29], “Traffic Lights” [30], “BOILED-Egg” [31], Egan rules [32], and Muegge rules [33]. Of note, some of the compounds are marked as likely promiscuous binders or aggregators due to matching Pan-assay interference compounds (PAINS) PAINS filters [34] or identity/high similarity to compounds present in the Aggregator Advisor database [35]. Curcumin is the only compound both explicitly listed as a known aggregator and matching PAINS filters, in accord with multiple reports of false positive results caused by curcumin in biological assays [36]. Some of the remaining xanthone derivatives, e.g., alvaxanthone, rheediaxanthone B, and rheediaxanthone C, match PAINS filters and are at least 70% similar to compounds known to act via aggregation. Therefore, more thorough future assays, especially ones focusing on enzyme activity in vitro, should involve measures testing for aggregation and promiscuous binding, e.g., dynamic light scattering (DLS) DLS measurements or activity assays with unrelated enzymes [37].

### 2.3. TS Inhibition

The molecular docking campaign resulted in 20 compounds (Table 1, compounds **1**–**20**) suggested as potentially selective inhibitors of nematode TS. Their activity against four enzyme preparations, corresponding to two mammalian (human and mouse, hTS and mTS, respectively) and two nematode species (*T. spiralis* and *C. elegans*, *Ts*TS and *Ce*Ts, respectively) was assessed by testing inhibition by each compound at three concentrations, i.e., 10, 100, and 1000 µM (unless low solubility did not allow to reach the highest concentration), of the enzyme-catalyzed reaction run with 20 µM dUMP and either 250 µM or 50 µM meTHF in the reaction mixture. The results pointed to alvaxanthone (Table 1, compound **15**) as the strongest inhibitor, showing the IC_50_ values with different enzyme preparation studied ranging from below 10 µM to around 100 µM, with further strong inhibitors in this group being rheediaxanthone B and rheediaxanthone C (Table 1, compounds **17** and **18**, respectively) but only for mTS. Other compounds inhibited TS by at least one order of magnitude weaker. Further comparison of inhibition by alvaxanthone of *Ts*TS and hTS, with 20 µM dUMP and 50 µM meTHF in the reaction mixture, showed the IC_50_ values of 41.9 ± 8.1 µM (3) and 6.3 ± 1.6 µM (3), respectively. Furthermore, initial results showed time-dependent inhibition, suggesting its slow-binding character [38]. Inhibition by rheediaxanthone B of *Ts*TS, *Ce*TS, hTS, and mTS, tested under the above conditions, showed the IC_50_ values of 50.8 µM, 11.4 µM, 17.9 µM, and 14.3 µM, respectively (the experiments have not been repeated). Compared to alvaxanthone, also α-mangostin, another xanthone derivative, characterized by a high structural similarity to alvaxanthone and demonstrated previously to inhibit *C. elegans* population growth [39], was not a rather strong inhibitor of TS (Table 1, compound **21**).

### 2.4. Toxicity to C. Elegans

Alvaxanthone was found to be also a relatively strong inhibitor of *C. elegans*, which is considered as a model of parasitic nematodes [14], causing reduction of growth of the nematode population with LC_50_ of 4.60 ± 0.57 µM (4). The latter value was similar to those previously found [39] for the above mentioned α-mangostin (3.8 µM) and the well-known antiparasitic drug mebendazole (4.0 µM), pointing to a potential antiparasitic activity of alvaxanthone.

Considering the inhibitory activities of alvaxanthone (Table 1, compound **15**) against both TS and the nematode population growth, several structurally similar compounds were selected, including 1,3-OH-2-OMe-xanthone, rheediaxanthone B and C, 1-OH-7-OMe-xanthone, and 2-OMe-xanthone (Table 1, compounds **16**–**20**), to be tested against *C. elegans*. Only rheediaxanthone B and 2-OMe-xanthone were active, showing LC_50_ values of 16.0 ± 10.2 µM (2) and 33.8 ± 2.1 µM (2), respectively, with the remaining derivatives demonstrating weaker effects by at least one order of magnitude.

### 2.5. Cytotoxicity

The same group of xanthones, including alvaxanthone, 1,3-OH-2-OMe-xanthone, rheediaxanthone B and C, 1-OH-7-OMe-xanthone, and 2-OMe-xanthone, underwent cytotoxicity tests. The compounds varied significantly in both toxicity and selectivity (Table 2). While the strongest toxicity was exerted by rheediaxanthone B and C and alvaxanthone, only alvaxanthone and rheediaxanthone B caused stronger inhibition of cancer (glioma cells U-118 MG) than normal (human fibroblasts-BJ) cells (Table 2). Therefore, these two compounds were selected for further studies. 

**Figure 1:**
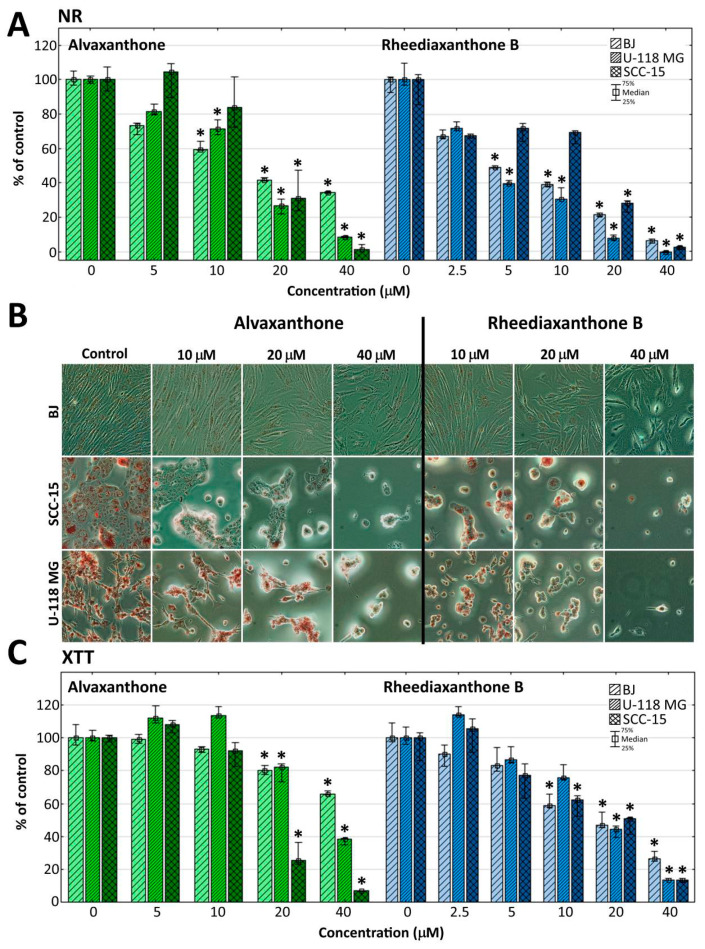
Viability of BJ, U-118 MG and SCC-15 cells after 48 h treatment with alvaxanthone or rheediaxanthone B, estimated by NR (**A**) and XTT (**C**) assays. Each bar represents the median of a population of 9 results, obtained in consequence of three independent experiments, each done in triplicate. The whiskers are the lower (25%) and upper (75%) quartile ranges. The results significantly different from control (in a view of the Kruskal–Wallis test) are marked * (*p* ≤ 0.05). (**B**) Cell morphology and neutral red accumulation following 48 h alvaxanthone or rheediaxanthone B treatment and 1 h incubation with neutral red. Red vesicles are lysosomes containing the dye.

Biological effect of alvaxanthone and rheediaxanthone B on normal (BJ) and cancer cells (U-118 MG and SCC-15) was estimated using two viability assays, the neutral red uptake (NR) and tetrazolium salts reduction (XTT) assays. The results of three independent triplicate assays are presented in Figure 1.

Both assays revealed concentration- and cell type-dependent toxicity of each of the two compounds. Tested by the more sensitive NR assay, rheediaxanthone B was slightly more toxic (IC_50_ < 10 µM, compared to alvaxanthone (15 µM < IC_50_ < 18 µM), none of them showing a significant selectivity against cancer cells. At the highest concentration of 40 µM, cancer cells were distinctly more susceptible than normal cells to alvaxanthone (viability decreased to 10% with U-118 MG, and 1.5% with SCC-15 cells, vs. 35% with fibroblasts). On the other hand, activity of mitochondrial dehydrogenases and oxidoreductases, tested by the XTT assay, provided information about the condition of mitochondrial metabolism and indicated selectivity of alvaxanthone against both SCC-15 and U-118 MG cells (compared to BJ fibroblasts 5.3-fold and 2.7-fold more sensitive, respectively; Figure 1). Thus, alvaxanthone may be damaging mitochondria and impairing their metabolism exerting one of possible mechanisms active in destroying cancer cells [40]. Changes of cell morphology in dependence on increasing alvaxanthone and rheediaxanthone B concentration were also recorded. While the glioma and squamous carcinoma cells formed clusters, lost their adhesion, shrank, and accumulated less NR dye, normal fibroblasts preserved rather normal shapes but the amount of the neutral red dye and confluence of cells decreased. In addition, a lower number of BJ cells was detected (Figure 1B).

A possible cytotoxic mechanism of alvaxanthone can be hypothesized, relying in glioma and fibroblasts on cellular and lysosomal membrane disruption rather than mitochondrial function impairment, as indicated by lower viability of both U-118 MG and BJ cells observed in the NR than XTT assay at the highest alvaxanthone concentration. A different toxic pattern was observed in squamous carcinoma cells, manifested by similar cell viability levels at the whole range of alvaxanthone concentrations and equal IC_50_ values observed with both assays (IC_50_ = 17 μM), indicating both cell membrane damage and mitochondrial dysfunction. These results suggest also that alvaxanthone impact on mitochondrial metabolism was in SCC-15 cells stronger compared to the other cell lines. In accord, Hou et al. [41] showed the IC_50_ values estimated for human oral squamous carcinoma cells (HSC-2 cell line) and normal human gingival fibroblasts (HGF) after 24 h incubation to be almost equal (22 µM and 25 µM, respectively).

### 2.6. Proliferation

**Figure 2:**
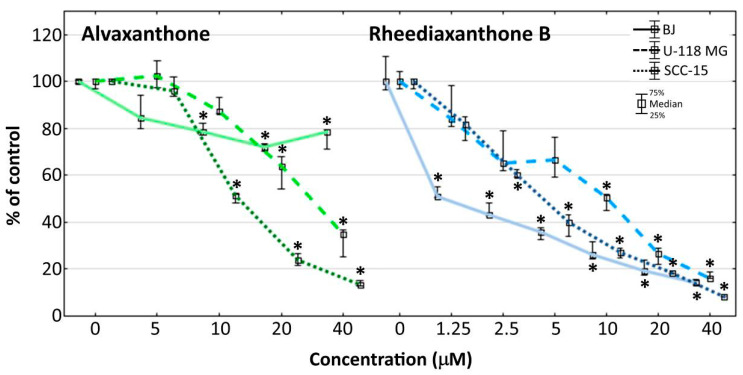
Proliferation of cancer (U-118 MG and SCC-15) and normal (BJ) cells after 72 h incubation with 5–40 μM alvaxanthone or 1.25–40 μM rheediaxanthone B, determined with the CyQUANT GR Cell Proliferation Assay Kit. Median results are presented of three independent experiments, each performed in triplicate. The whiskers are lower (25%) and upper (75%) quartile ranges. The results significantly different from control (in view of the Kruskal–Wallis test) are indicated * (*p* ≤ 0.05). Images collected with Olympus IX-83 microscope with contrast phase (scale bar = 100 µm).

The influence of alvaxanthone and rheediaxanthone B on cell proliferation (Figure 2) was assessed by quantitative measurement of the total cellular DNA content, proportional to the cell number [42].

Alvaxanthone was a somewhat stronger inhibitor of the cancer (glioma and squamous carcinoma) than normal (BJ fibroblasts) cell proliferation, the effect noticeable only at concentrations above 10 μM, with the inhibition at 40 μM alvaxanthone being by 5.8- and 2.3-fold weaker with normal (BJ) than cancer (SCC-15 and U-118 MG, respectively) cells (Figure 2). Rheediaxanthone B, being apparently a stronger proliferation inhibitor than alvaxanthone, shows also similar selectivity but at a lower concentration range (up to some 2 μM; Figure 2).

### 2.7. Adhesion

**Figure 3:**
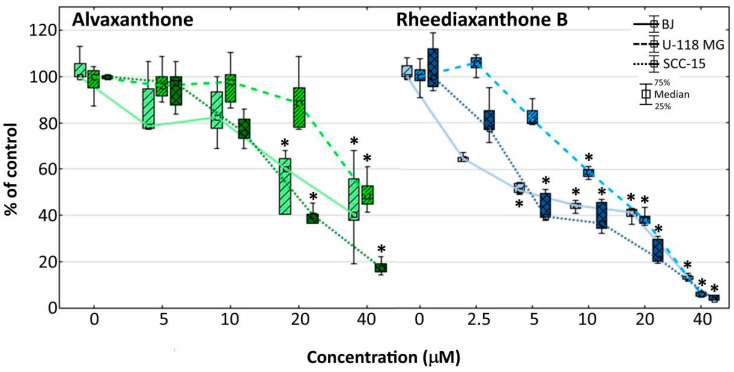
Influence of alvaxanthone or rheediaxanthone B on BJ, U-118 MG, and SCC-15 cells adhesion after 48-h treatment. The crystal violet assay was used. Results are medians of triplicate assays of three independent experiments expressed as a % of non-treated controls. The whiskers are lower (25%) and upper (75%) quartile ranges. * *p* ≤ 0.05, Kruskal–Wallis test (against non-treated control).

Cancer metastasis, resulting from an impairment of adhesion between cancer cells and the extracellular matrix may cause cell migration from a primary tumor into distant parts of an organism [43]. The cristal violet assay was used to examine alvaxanthone and rheediaxanthone B ability to influence cellular adhesion. The results indicate alvaxanthone significantly reduces adhesion of normal fibroblasts and squamous carcinoma cells, compared with glioma cells, at lower (20 µM vs. 40 µM) concentration (Figure 3).

**Figure 4:**
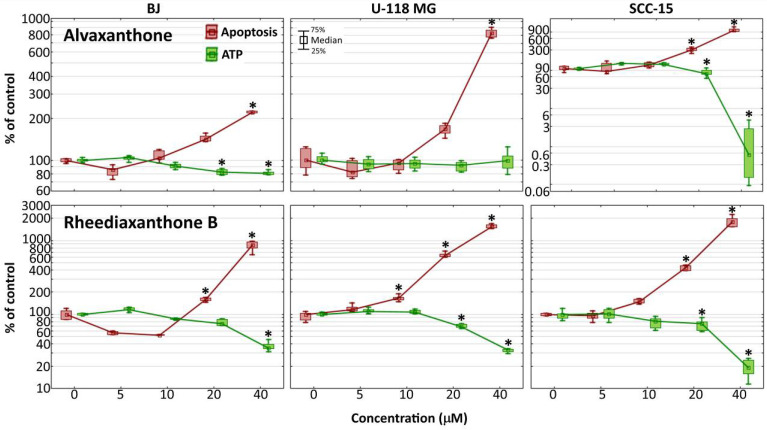
Changes of caspase-3/7 activities and intracellular ATP level in BJ, U-118 MG, and SCC-15 cells after 48-h incubation with 5–40 µM alvaxanthone or rheediaxanthone B. Median results are presented of three independent experiments, each performed in triplicate. The whiskers are lower (25%) and upper (75%) quartile ranges. The results significantly different from control (in view of the Kruskal–Wallis test) are indicated as * (*p* ≤ 0.05).

The effect of rheediaxanthone B was apparent at lower concentrations (at 5 µM with BJ and SCC-15, and 10 µM with U-118 MG cells; Figure 3). The results are consistent with those of the NR cytotoxicity assay (Figure 1), showing similar profile of the toxic effect with respect to the cell lines and drug concentrations affecting their growth, pointing to the decrease of cell adhesion to be caused by each of the two prenylated xanthones as a result of the compound’s toxic action, without increasing the probability of metastasis during therapy. Additional information provides Figure 4, presenting changes of the intracellular ATP level in treated cells. With both compounds at concentrations causing decreased cell adhesion, considerably depleted ATP levels were apparent that probably reduced cells capacity to move and migrate. Thus, considering potential chemotherapeutic application, the tested compounds should not be expected to increase migration of cancer cells to other parts of an organ or organism or lead to metastasis processes.

### 2.8. Caspase-3/7 and ATP Level

The Apo-ONE^®^ Homogenous Caspase-3/7 Assay provides a measurement of the executioner caspases 3/7 activation, characteristic for apoptosis induced via both the intrinsic and extrinsic pathway [44]. It was used to determine apoptosis induction in cells by alvaxanthone and rheediaxanthone B. In each of the three cell lines, compared to the respective non-treated control, treatment with each of the two compounds for 48 h led to a significant activation of the caspases 3/7 (by 2- to 10-fold for alvaxanthone and 8- to 18-fold for rheediaxanthone B), the effect being stronger in cancer than normal cells (Figure 4).

As induction of apoptosis has been previously suggested to cause a caspase-dependent fall of ATP levels [45,46], the CellTiterGlo^®^ Luminescent Cell Assay was performed to determine changes in ATP level. After incubation with alvaxanthone, the level of ATP in glioma cells remained unaltered; in normal fibroblasts, it showed only a slight decrease, and, in SCC-15, it was dramatically reduced. On the other hand, rheediaxanthone B caused a reduction of ATP level down to around 30% in glioma cells and fibroblasts, and to 20% in squamous carcinoma cells (Figure 4). In general, with increasing Caspase-3/7 activity the ATP level was decreasing, in accord with the results of Reference [46]. However, contrary to the latter, Oropesa et al. demonstrated that, during apoptosis in alvaxanthone-treated glioblastoma cells, the level of ATP was unchanged or even increased during early phases of apoptosis, despite caspase 3 and 7 undergoing activation [47].

### 2.9. Predicted Binding Mode of Alvaxanthone and Rheediaxanthone B

**Figure 5:**
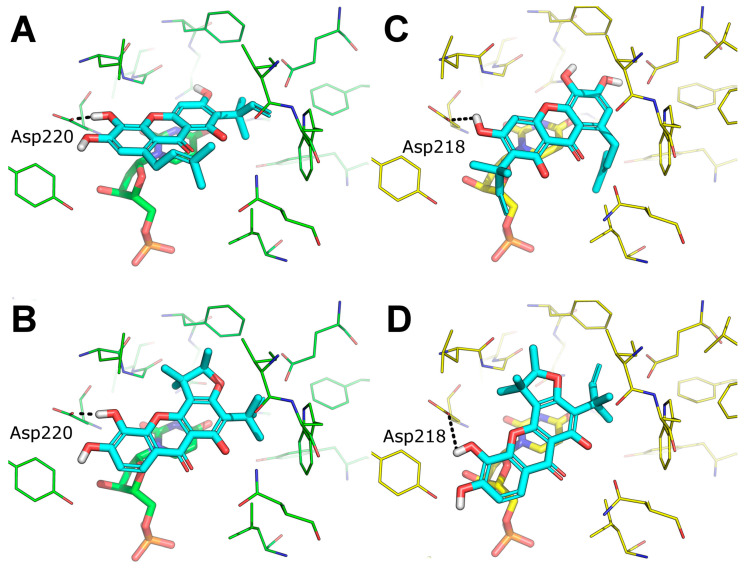
Predicted binding mode of alvaxanthone and rheediaxanthone against TS. (**A**,**B**) docking pose of alvaxanthone (**A**) and rheediaxanthone (**B**) against the catalytic site of TS from *C. elegans,* PDB ID 4IQQ (green lines). (**C**,**D**) docking pose of alvaxanthone (**C**) and rheediaxanthone (**D**) against the catalytic site of TS from homo sapiens PDB ID 5X5D (yellow lines). TS inhibitors are shown as cyan lines. dUMP is shown as sticks and colored as the protein. H-bonds are showed as black dashed lines. Only residues within 5 Å from the docked ligands are shown.

The possible binding mode of the most interesting TS inhibitors identified by virtual screening and in vitro tests, i.e., alvaxanthone and rheediaxanthone B, was investigated by molecular docking against human and *C. elegans* TS. Compared to the virtual screening settings, here, we stored a larger number of binding poses of each compound to enrich our analysis with statistical significance. Overall, both compounds proved to bind within the TS catalytic site, in close proximity and in stacking conformation to the co-crystallized dUMP cofactor. Notably, both compounds adopted a very similar binding mode against the catalytic site of TS from both human and *C. elegans*, which is not surprising according to the high degree of sequence and structural identity between TS from the two species [48]. Besides stacking to dUMP, the major anchor point is the side chain of Asp220 (in *C. elegans* Ts) or Asp 218 (in human TS), which is H-bonded to a hydroxyl group connected to the xanthone ring (Figure 5). Similar to docking poses, also docking scores highlighted that both alvaxanthone and rheediaxanthone B bind with comparable affinity to each protein (chemgauss4 score for alvaxanthone: −8.60 (*C. elegans*) and −8.91 (human); rheediaxanthone: −8.10 (*C. elegans*) and −8.50 (human)). These theoretical results are in agreement with experimental data above and may facilitate the design of TS inhibitors with improved features.

## 3. Discussion

The present results highlight two natural compounds selected by virtual screening of an in-house library of natural products and their derivatives as inhibitors of the reaction catalyzed by TS of different species. The most potent enzyme inhibitors are between xanthone derivatives, with alvaxanthone inhibiting stronger than rheediaxanthone B and the latter stronger than rheediaxanthone C. Similarly, another commercially available compound, α-mangostin (Table 1, compound **21**), which is a close analogue of alvaxanthone [39], is as potent inhibitor as the latter (Table 1). Comparison of the xanthone group (Table 1, compounds **15**–**21**) with regard to the inhibitory potential allows a rough identification of the structural requirements, including hydroxyls at the positions 1, 3, and 6, and hydrophobic substituents at the positions 2 and 8.

The xanthone derivatives capable of inhibition of TS-catalyzed reaction, including alvaxanthone, α-mangostin, and rheediaxanthone B, are toxic for *C. elegans* and mammalian cells. However, rheediaxanthone C that inhibits the enzyme does not show toxicity for the worm. Besides, 2-OMe-xanthone, lacking an apparent TS inhibitor activity, is toxic only for *C. elegans* (Table 1 and Table 2 [39]). Thus, it remains to be established whether TS is the target in both the organismal (*C. elegans*) and cell-based model systems.

Of particular interest is nematocidal activity of alvaxanthone, which proved to be toxic for *C. elegans* to a similar extent as the well-known reference anthelmintic drug mebendazole [49,50] and α-mangostin [39]. While the lack of selective toxicity against the nematode worms versus human cells does not point to alvaxanthone as a potential chemotherapeutic drug to combat human nematode parasites, it does not preclude its potential application against plant nematode parasites. Besides, it may be treated as a leading compound to be modified in search of a selective chemotherapeutic drug. Furthermore, as the compound is also a TS inhibitor, it may be used as a tool in further studies of the physiological significance of the high expression of TS in nematodes’ cells (see Introduction).

Alvaxanthone has been previously demonstrated to show antimicrobial [51,52], antifungal [53], and cytotoxic [41,53] activities. To the best of our knowledge, there are no records on the biological activity of rheediaxanthone B and rheediaxanthone C. The present results show alvaxanthone, rheediaxanthone B and rheediaxanthone C to be cytotoxic. Compared to alvaxanthone, the cytotoxic and antiproliferative effects of rheediaxanthone B were slightly stronger and associated with lowered adhesion and elevated activities of the executioner caspases (Figure 1, Figure 2, Figure 3 and Figure 4). While in glioblastoma and fibroblast cells alvaxanthone toxicity was based mostly on disturbing lysosomal membranes or alterations in cellular membrane, in the SCC-15 cells, it was associated with both cell membrane and mitochondrial metabolism changes. Of particular interest is an antiproliferative effect of alvaxanthone in tumor cells, associated by a selective toxicity against mitochondria observed in cancer cells and cell death via the more desirable apoptosis pathway. Of note is also that in view of lowered adhesion being caused by each of two compounds toxicity (Figure 1 and Figure 3), neither alvaxanthone nor rheediaxanthone B appear to increase the probability of metastasis. Overall, these natural products share common pharmacophoric features and stand as a promising starting point for the design of further generation of TS inhibitors inspired by natural sources.

## 4. Materials and Methods

### 4.1. Materials 

The compounds selected to be tested were obtained from the in-house library of natural compounds organized and maintained by the group of Professor *Bruno Botta* of Sapienza University of Rome. In total, 865 compounds that composed the in-house library of natural products at the time of the study were screened in silico by molecular docking simulations. We used the above-mentioned library as a source of natural compounds endowed with high structural and chemical diversity, with the aim to identify novel chemotypes of TS inhibitors. α-Mangostin (**21**) was purchased from Sigma-Aldrich (St. Louis, MO, USA). Standard samples of compounds **1**, **2**, **3**, **4**, **6**, and **9** were purchased from Sigma-Aldrich (St. Louis, MO, USA) and compared with the corresponding samples from the library. The compounds tested with cell cultures were prepared from stock solutions in corresponding cell culture media by adjusting the DMSO concentration to ≤ 0.05% (showing no significant effect on treated cells).

### 4.2. Computational Methods

The in-house library of natural products available at the Department of Chemistry and Technology of Drugs of Sapienza University of Rome (Prof. Bruno Botta) was computationally screened against the crystallographic structure of C. elegans TS in complex with dUMP and a small molecule inhibitor (PDB ID: 4IQQ) through molecular docking simulations. Docking was carried out with the FRED program from OpenEye [54,55], using the default settings and the highest docking resolution. Ligand conformational analysis was carried out with OMEGA from OpenEye [56,57], storing up to 600 conformers for each ligand. In docking against the human form of TS, the PDB ID: 5X5D (TS in complex with dUMP) was used [58]. Receptor structures were prepared with the make_receptor utility of FRED. In virtual screening, only the top-scoring pose of each ligand was stored and analyzed, while, in docking alvaxanthone and rheediaxanthone B, up to 20 docking poses were stored. Druglikeness and ADME/tox properties of the compounds were assessed using freely available online tools: FAF-Drugs4 [59], SwissADME [60], and Aggregator Advisor [35].

### 4.3. Chemistry

Standard samples of compounds **1**, **2**, **3**, **4**, **6**, **8**, and **9** were purchased from Sigma-Aldrich (St. Louis, MO, USA) and compared with the corresponding samples from the library. Samples of compounds **1**, **2**, **3**, **4**, **6**, **8**, and **9** from the library resulted to be spectroscopically and chromatographically identical to the commercial samples.

Compound **5** showed NMR spectra identical to those reported in the literature [61,62,63]. Compound **7** showed NMR spectra in accordance with those reported in the literature [64].

NMR spectra identical to those reported in the literature were observed also for compounds **10** [65], **11** [66], **12** [67], **13** [68], **14** [69,70], **15** [71], **16** [72,73], **17** [74], **18** [74], **19** [75], and **20** [75].

### 4.4. TS preparation

The recombinant human, rat, mouse, *T. spiralis* and *C. elegans* TS proteins were produced, purified and separated into phosphorylated and non-phosphorylated fraction, as previously described [20]. TS activity was monitored, following tritium release from [5-^3^H]dUMP, and its inhibition studied as earlier reported [76]. At concentrations ≤10% DMSO (used to dissolve compounds to be tested) did not affect the reaction rate.

### 4.5. Determination of Toxicity to C. elegans, a Nematode Parasite Model

*C. elegans* was maintained as previously described [17]. The effect of the compounds studied on *C. elegans* population growth was determined as earlier presented [39].

### 4.6. Cell Culture Studies

Human glioblastoma cells U-118 MG (ATCC^®^ HTB-15), human squamous carcinoma cells SCC-15 (ATCC^®^ CRL-1623), and normal human skin fibroblasts (ATCC^®^ CRL-2522) were grown, their morphology checked, and number and viability estimated as earlier presented [39].

Influence of selected compounds on cells was tested by performing the cytotoxicity (NR and XTT) assays, proliferation assay, cell adhesion assay, cell migration assay, determination of intracellular ATP level, and apoptosis assay as previously reported [39].

### 4.7. Statistical Analysis

This was performed using the nonparametric Kruskal-Wallis test to estimate the differences between the compounds-treated cells and non-treated control in each cell lines. *p* ≤ 0.05 was considered as statistically significant. Calculations were performed using Statistica 12 (StatSoft, Tulsa, OK, USA). The results describing TS inhibition and reduction of *C. elegans* population are presented as means ± S.E.M., or means ± % difference between the mean and each of two results, followed by the number of experiments (N) in parentheses.

## Figures and Tables

**Table 1:** Assessment of the IC_50_ values describing inhibition of thymidylate synthases (TSs) of different origin by compounds selected by the 3D structure-based virtual search of the in-house library of natural compounds (Table 1, compounds **1**–**20**) and obtained from that library, and by α-mangostin (Table 1, compound **21**), a close structural analogue of alvaxanthone, included in the study after learning inhibitory properties of the latter and purchased from a commercial source.

Compound (No.)	Structure	IC_50_ [µM] Assessment (% Remaining Activity) ^a^			
		mTS	hTS	*Ce*TS	*Ts*TS
Curcumin (**1**)	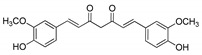	>50(83) <500(37)	>50(86) <500(20)	>50(84) <500(30)	>500(73)
Aloin (**2**)	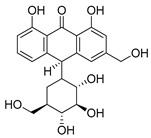	>1000(95)	>100(94) <1000(49)	>100(83) <1000(36)	>1000(93)
Chlorogenic acid (**3**)	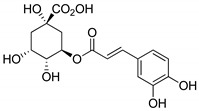	>1000(83)	>1000(76)	>1000(82)	>1000(94)
Phloretin (**4**)	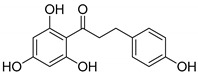	>100(78) <1000(45)	>100(86) <1000(31)	>100(82) <1000(48)	>1000(61)
Sophoronol (**5**)	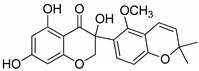	>100(75) <1000(10)	>100(75) <1000(8)	>100(77) <1000(8)	>100(74) <1000(35)
Bixin (**6**)	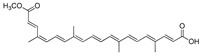	>667(73)	(667)NI ^b^	>67(93) <667(46)	>67(76) <667(47)
Myricetin-4′-OAc (**7**)	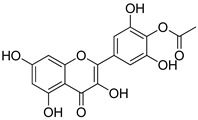	>100(61) <1000(49)	>100(56) >1000(31)	>100(69) <1000(28)	>1000(66)
Agnuside (**8**)	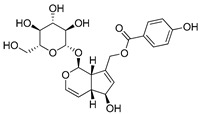	>1000(72)	>100(90) <1000(47)	>100(100) <1000(36)	>1000(63)
Clusiacitran A (**9**)	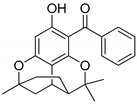	>1000(62)	>100(89) <1000(47)	>100(88) <1000(44)	>1000(95)
Jatrowediol (**10**)	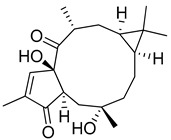	>1000(80)	>100(100) <1000(43)	>1000(77)	>1000(95)
6-Chromane-*O*-7-flavanone (**11**)	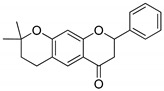	>100(91) <1000(10)	>10(100) <100(37)	>10(100) <100(24)	>1000(85)
Alnusin (**12**)	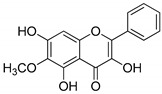	(1000)NI ^b^	>100(85) <1000(30)	>1000(80)	(1000)NI ^b^
Leucosceptoside A (**13**)	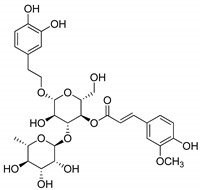	>1000(77)	>1000(66)	>1000(83)	(1000)NI ^b^
(±)-7-Hydro-xy-8-C-prenyl flavanone (Ovaliflavanone B) (**14**)	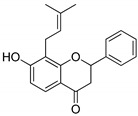	>100(71) <1000(2)	>100(74) <1000(7)	>100(65) <1000(3)	>100(90) <1000(25)
Alvaxanthone (**15**)	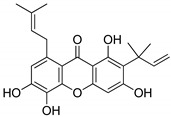	>10(79) <100(40)	<10(39)	>10(85) <100(18)	>10(98) <100(47) >10 ^†^(97) <100 ^†^(14)
1,3-OH-2-OMe-Xanthone (**16**)	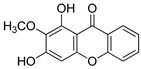	>100(67) <1000(32)	>100(54) <1000(27)	>1000(65)	>100(92) <1000(23) >100 ^†^(82) <1000 ^†^(32)
Rheediaxanthone B (**17**)	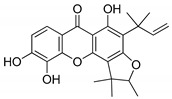	>10 ^†^(56) <100 ^†^(2)	>10 ^†^(59 <100 ^†^(5)	>10 ^†^(59) <100 ^†^(14)	>10 ^†^(68) <100 ^†^(40)
Rheediaxanthone C (**18**)	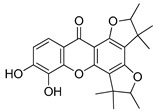	>10 ^†^(77) <100 ^†^(39)	>100 ^†^(57)	>100 ^†^(71)	>100 ^†^(73)
1-OH-7-OMe-Xanthone (**19**)	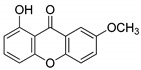	>1000 ^†^ (94)	>1000 ^†^ (100)	>1000 ^†^ (81)	>1000 ^†^ (89)
2-OMe-Xanthone (**20**)	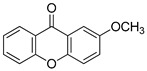	>1000 ^†^ (84)	>1000 ^†^ (88)	>1000 ^†^ (95)	>1000 ^†^ (92)
α-Mangostin (**21**)	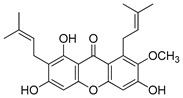	>10 ^†^(75) <100 ^†^(5)	>10 ^†^(89) <100 ^†^(19)	>10 ^†^(79) <100 ^†^(27)	>100 ^†^(85)

^a^ Inhibition by each compound was tested at three concentrations in the enzyme-catalyzed reaction run with 20 µM deoxyuridine monophosphate (dUMP) and either 250 µM or 50^†^ µM methylenetetrahydrofolate (meTHF) in the reaction mixture. The remaining activity (%) at an indicated concentration is shown in parentheses following that concentration. ^b^ No inhibition. The highest obtained concentration of a compound (reported in parentheses) showed no inhibition of the activity of a specific TS variant. ^†^ The reaction run at 20 µM dUMP and 50 µM meTHF in the reaction mixture.

**Table 2:** Cytotoxicity of alvaxanthone and several other xanthones determined by the neutral red assay.

Compound (No.)	IC_50_ (μM) ^a^		
	BJ	U-118 MG	SCC-15
Alvaxanthone (**15**)	15.76	13.07	17.66
1,3-OH-2-OMe-xanthone (**16**)	>40	>40	>40
Rheediaxanthone B (**17**)	5.5	4.75	9.2
Rheediaxanthone C (**18**)	5.95	18.27	11.04
1-OH-7-OMe-Xanthone (**19**)	>40	40	>40
2-OMe-Xanthone (**20**)	>40	>40	>40

^a^ Calculated by plotting the median cell viability (% of control) values against compound concentrations (μM), followed by abscissa transformation to the logarithmic form. The logarithmic regression led to the y = aln(x) + b equation, allowing to calculate the IC_50_ value.
